# Influence of parity on weight gain during pregnancy in women with Gestational Diabetes: A retrospective cohort study

**DOI:** 10.12669/pjms.41.1.11412

**Published:** 2025-01

**Authors:** Lin Lin, Ruiyun Chen, Haifeng Lin, Ting Yao, Zihua Chen, Lianghui Zheng

**Affiliations:** 1 Lin Lin Fujian Maternity and Child Health Hospital, College of Clinical Medicine for Obstetrics, Gynecology and Pediatrics, Fujian Medical University. P.R. China; 2 Ruiyun Chen Fujian Maternity and Child Health Hospital, College of Clinical Medicine for Obstetrics, Gynecology and Pediatrics, Fujian Medical University. P.R. China; 3 Haifeng Lin Fujian Maternity and Child Health Hospital, College of Clinical Medicine for Obstetrics, Gynecology and Pediatrics, Fujian Medical University. P.R. China; 4 Ting Yao Fujian Medical University, Fuzhou, Fujian Province 350001, P.R. China; 5 Zihua Chen Fujian Maternity and Child Health Hospital, College of Clinical Medicine for Obstetrics, Gynecology and Pediatrics, Fujian Medical University. P.R. China; 6 Lianghui Zheng Fujian Maternity and Child Health Hospital, College of Clinical Medicine for Obstetrics, Gynecology and Pediatrics, Fujian Medical University. P.R. China

**Keywords:** Gestational diabetes mellitus, Gestational weight gain, Maternal complications, Neonatal complications, Parity

## Abstract

**Objective:**

This retrospective cohort study aimed to investigate the effects of parity on gestational weight gain (GWG) and its association with maternal and neonatal outcomes in women with gestational diabetes mellitus (GDM).

**Methods:**

This retrospective cohort study data from 2,909 pregnant women with GDM who delivered between 2021 and 2023 at Fujian Maternity and Child Health hospital, were analyzed. Participants were categorized into nulliparous (no previous births), primiparous (one previous birth), and multiparous (two or more previous births) groups. Women aged 18–45 years with singleton pregnancies were included, while those with multiple pregnancies, pre-existing diabetes, hypertension, thyroid disorders, or incomplete BMI data were excluded. Data extracted from hospital records included demographic information, BMI, GWG at various gestational stages, and pregnancy outcomes. Statistical analysis involved logistic regression models, adjusting for confounders such as maternal age, BMI, and gestational age at delivery. P-values < 0.05 were considered significant.

**Results:**

Primiparous and multiparous women exhibited significantly higher GWG than nulliparous women (p < 0.01). Primiparous women had reduced risks of pregnancy-induced hypertension (adjusted OR, 0.64; 95% CI, 0.45–0.92) and premature rupture of membranes (adjusted OR, 0.58; 95% CI, 0.48–0.70). Both primiparous and multiparous women had lower risks of delivering large-for-gestational-age newborns or those with neonatal jaundice.

**Conclusion:**

Parity significantly influences GWG and maternal/neonatal outcomes in GDM pregnancies, highlighting the need for parity-specific guidelines.

## INTRODUCTION

Gestational diabetes mellitus (GDM) manifests during pregnancy, affecting approximately 7% of pregnancies globally, although the prevalence can be significantly higher depending on the population studied.^1^,^2^ GDM is characterized by glucose intolerance that is first recognized during pregnancy and poses significant risks for both maternal and neonatal health.^3^ For the mother, GDM increases the risks of cesarean delivery, preeclampsia, and future development of Type-2 diabetes mellitus.^4^,^5^ For the fetus, GDM is associated with risks such as macrosomia (excessive birth weight), neonatal hypoglycemia, and an increased likelihood of glucose intolerance and overweight in childhood and adulthood.^6^,^7^

One of the critical factors in managing GDM is controlling the gestational weight gain (GWG).^8^ The Institute of Medicine provides guidelines on the optimal weight gain during pregnancy based on the pre-pregnancy body mass index (BMI), but these recommendations may not be sufficient due to other factors that influence GWG, such as parity.^9^ Parity, defined as the number of previous pregnancies a woman has had, can significantly impact both the trajectory of weight gain during pregnancy and pregnancy outcomes.^10^–^12^ Higher parity has been associated with greater GWGs, potentially due to physiological changes accumulating with each subsequent pregnancy.^13^,^14^ Additionally, multiparous women (those who have had one or more previous pregnancies) may experience different metabolic regulation and fat deposition patterns than those in nulliparous women (those on their first pregnancy).^15^,^16^ Understanding the association between parity and GWG is particularly important in women with GDM, as these women have increased risks for adverse outcomes, and excessive weight gain can exacerbate these risks.^17^,^18^

Particularly in the context of GDM, monitoring the GWG is important. However, whether parity influences GWG and subsequent pregnancy outcomes in this population remains unclear. Most studies on GDM focus on the role of glycemic control and general risk factors without adequately considering the impact of parity on weight gain and pregnancy outcomes.^19^ Given the potential for parity to modify the association between pregnancy outcomes and the GWG, investigating this interaction thoroughly is crucial.^20^ We designed this study to assess the potential associations between GWGs and parity in women with GDM and determine whether these variations impact pregnancy outcomes such as pregnancy-induced hypertension, mode of delivery, preterm birth, birth weight, and neonatal outcomes.

## METHODS

We conducted this retrospective cohort study with data from the comprehensive database between 2021 and 2023 at Fujian Maternity and Child Health hospital, containing extensive details on pre-pregnancy health examinations and antenatal care. The study was designed to analyze the potential associations between parity, GWG, and pregnancy outcomes in women diagnosed with GDM. The detailed records of the hospital’s database enabled robust tracking of neonatal and maternal health outcomes.

### Ethical approval:

The institutional Medical Ethics Committee approved the study (approval number 2024KY200), Dated August 14, 2024) which was conducted ensuring adherence to ethical standards, including patient confidentiality and personal data protection. The Ethics Committee granted an exemption from requiring informed consents given our study’s retrospective nature.

### Study Population:

The study was conducted with records of 2,909 pregnant women with GDM who delivered between 2021 and 2023 at Fujian Maternity and Child Health hospital and met the inclusion criteria.

### Inclusion Criteria:

- Pregnant 18 to 45 years old women.
- Singleton pregnancies.
- GDM diagnosis based on the 75 g oral glucose tolerance test (OGTT) administered between gestational weeks 24 and 28.
- Availability of complete clinical records.

### Exclusion Criteria:

- Multiple pregnancies (twins, triplets, etc.).
- Spontaneous miscarriage or stillbirth.
- Missing or incomplete data.

### Data Collection:

We extracted data from the hospital’s electronic medical records using unique personal identification numbers to track each participant’s medical history accurately. The database contained data providing a comprehensive overview of maternal and neonatal outcomes, from pre-pregnancy to postnatal follow-ups. We systematically organized the collected data into an Excel spreadsheet for further analysis.

The maternal characteristics included age, gravidity (number of pregnancies), parity (number of births), preterm history, height, pre-pregnancy weight, weight at delivery, and GWG. Pregnancy outcomes included cesarean-section; preterm birth (birth before completion of 37 gestational weeks), post-term pregnancy (birth after completion of 42 gestational weeks), pregnancy-induced hypertension (PIH) (at least two instances of systolic blood pressure ≥ 140 mmHg and/or diastolic blood pressure ≥ 90 mmHg four or more hours apart and after 20 weeks of gestation); gestational diabetes mellitus (GDM) (diagnosis based on a positive OGTT between the 24^th^ and 28^th^ gestational weeks); hypothyroidism or hyperthyroidism; abruption of placenta; and premature rupture of membranes (PROM).

Infant characteristics included sex, low birth weight (LBW) (< 2500 g), macrosomia (> 4000 g), gestational weeks at birth, small-for-gestational-age (SGA) (birth weight below the 10^th^ percentile of mean weight corrected for fetal sex and gestational age); large-for-gestational-age (LGA) (birth weight above the 90^th^ percentile of mean weight corrected for fetal sex and gestational age); pre-term birth; neonatal jaundice; NICU admission; asphyxia; and chorioamnionitis.

### Definition of variables:

We defined parity^21^ as the number of previous pregnancies carried to a viable gestational age (≥28 weeks) categorized as nulliparous (absence of previous pregnancies carried beyond 28 weeks), primiparous (one previous pregnancy carried beyond 28 weeks), and multiparous (two or more previous pregnancies carried beyond 28 weeks). We used these parity categories to investigate the influence of the number of previous pregnancies on the GWG and pregnancy outcomes. We used data from the nulliparous group as the reference for comparative analyses.

### Statistical Analysis:

We organized the data in Microsoft Excel sheets. We expressed normally-distributed continuous variables as means ± standard deviations and analyzed them using one-way ANOVA and Tukey’s post hoc test. For non-normally–distributed continuous variables, we presented data as medians with interquartile ranges (25^th^–75^th^ percentile) and analyzed them using the Kruskal–Wallis test. We conducted all statistical tests using SPSS version 25.0 (SPSS). Our multivariate logistic regression models included cumulative and multinomial logistic regression to adjust for confounders and determine independent risk factors for maternal and neonatal complications. We considered *p*-values lower than 0.05 for any test as reflecting statistical significance.

## RESULTS

We analyzed data of 2,909 women with GDM pregnant between 2021 and 2024 categorized into three parity groups, 1343 women were nulliparous (46.16%), 1494 primiparous (51.30%), and 72 multiparous (2.47%).

### Demographic and GWG Characteristics:

The distribution of demographic variables and GWG across different parity groups among pregnant women with GDM is presented in Table-I. We found significant differences in age, BMI, and GWG across these groups.

**Table-I:** Distribution of demographic variables and gestational weight gain in pregnant women with GDM.

Variables	Nulliparous (n = 1343)	Primiparous (n = 1494)	Multiparous (n = 72)	p-value
***AGE (years)***				
20-30	26.85(25.36-28.34)	27.00(25.44–28.56)	26.27(24.65–27.89)	<0.01*
30-40	32.63(30.34-34.91)	34.49(31.84–37.14)	34.04(31.38–36.70)	
40-50	41.13(39.55–42.71)	41.32(39.82–42.82)	41.60(39.76–43.44)	
***BMI***				
Underweight	17.55(16.76–18.34)	17.60(16.84–18.36)	17.52(16.71–18.33)	<0.01*
Normal	21.23(19.51–22.96)	21.62(19.94–23.20)	21.68(20.03–23.33)	
Overweight/Obese	27.33(25.09–29.57)	27.16(25.10–29.23)	28.61(25.90–32.26)	
***GESTATIONALWEIGHT***				
Weight at 12 weeks (kg)	56.20(47.30–65.10)	57.98(49.40–66.56)	57.05(47.72–66.38)	<0.01*
Weight at 16 weeks (kg)	57.00(48.14–65.87)	58.71(50.23–67.19)	57.65(49.27–66.03)	<0.01*
Weight at 20 weeks (kg)	58.72(49.49–67.95)	60.65(43.86–77.44)	59.07(50.89–67.25)	<0.01*
Weight at 24 weeks (kg)	61.19(52.48–69.90)	62.43(54.23–70.62)	61.65(53.65–69.64)	<0.01*
Weight at 28 weeks (kg)	62.96(54.33–71.59)	64.17(55.99–72.35)	63.26(55.21–71.31)	<0.01*
Weight at 32 weeks (kg)	64.46(55.79–73.13)	65.63(57.45–73.81)	64.68(56.85–72.52)	<0.01*
Weight at 36 weeks (kg)	66.26 (57.53–75.00)	67.20 (59.02–75.39)	65.88(57.94–73.82)	0.01*
***GESTATIONAL WEIGHT GAIN (GWG)***				
Weight gain at 12 weeks (kg)	1.00 (0–2.30)	1.10 (0–2.77)	1.05 (0.65–2.46)	0.01*
Weight gain at 16 weeks (kg)	2.00 (0.50–3.40)	2.02 (0.70–3.80)	2.30 (1.05–4.25)	0.02*
Weight gain at 20 weeks (kg)	3.70 (2.00–5.30)	3.75 (2.15–5.50)	3.77 (2.42–5.98)	0.44
Weight gain at 24 weeks (kg)	6.30 (4.50–8.00)	6.00 (4.10–8.00)	6.50 (4.25–7.90)	0.30
Weight gain at 28 weeks (kg)	8.00 (6.00–10.05)	7.80 (5.50–10.00)	8.50 (6.00–10.00)	0.37
Weight gain at 32 weeks (kg)	9.60 (7.20–11.75)	9.20 (6.95–11.60)	9.52 (7.30–11.31)	0.33
Weight gain at 36 weeks (kg)	11.26 (8.75–13.94)	10.83 (8.15–13.30)	10.77 (8.29–12.48)	<0.01*
Weight at delivery (kg)	67.83(58.86–76.80)	67.95(59.49–76.42)	66.63(59.04–74.21)	0.36
GWG	12.91 (8.25–17.57)	11.66(7.17–16.14)	11.60 (7.06–16.13)	<0.01*
GWG before OGTT (kg)	8.04 (4.63–11.45)	7.88 (4.35–11.41)	8.23 (4.57–11.90)	0.24
GWG after OGTT (kg)	4.87 (2.05–7.70)	3.78 (0.83–6.72)	3.36 (0.33–6.40)	<0.01*
Gestational week of OGTT (weeks)	26.04(24.89–27.18)	26.09(24.92–27.27)	25.94(24.75–27.12)	0.28

BMI: Body mass index; GWG: Gestational weight gain; OGTT: Oral glucose tolerance test,

* p <0.05.

### Age Distribution:

- Women aged 20 to 30 years showed had similar age distributions across parity groups.
- For the age group of 30 to 40 years. Primiparous women tended to be older than the rest, they had a mean age of 34.49 years compared to the mean of 32.63 years in nulliparous women and 34.04 years in multiparous women.
- Women aged 40 to 50 years were similarly aged across all parity groups, without significant differences noted.

### BMI Distribution:

- We found significant BMI distribution changes across parity groups (*p* < 0.01).
- Underweight women had similar BMI values across all parity groups.
- Normal BMI values were slightly higher in primiparous and multiparous women compared to those in nulliparous women.
- Overweight/obese women had higher BMI values in the multiparous group, indicating a trend toward increased BMI with higher parity.

### Gestational Weight Gain:

- Weights at various gestational weeks (12, 16, 20, 24, 28, 32, and 36 weeks) were consistently higher in primiparous women compared to those in nulliparous and multiparous women, with significant differences across all time points (*p* < 0.01).
- Total GWGs and weight gains at specific gestational weeks also varied significantly among the groups, with nulliparous women generally gaining more weight than primiparous or multiparous women. For instance, at 12 weeks, nulliparous women gained less weight than primiparous and multiparous women (*p* = 0.01).
- GWGs before and after the OGTT also differed across groups, with nulliparous women gaining significantly more weight post-OGTT than the women in the other groups (*p* < 0.01).

### Maternal and Neonatal Complications:

The analysis of maternal and neonatal complications revealed several significant findings related to parity (Table-II).

**Table-II:** Associations between parity and maternal/neonatal complications. Parity was adjusted for maternal age, BMI, GWG, and gestational weight at delivery.

Outcomes	Nulliparous (n = 1343)	Primiparous (n = 1494)	Multiparous (n = 72)
***Maternal Complications***			
***PIH n (%)***			
Unadjusted OR (95% CI)	Ref	0.78 (0.57–1.07)	0.19 (0.02–1.42)
Adjusted OR (95% CI)	Ref	0.64 (0.45–0.92) *	0.16 (0.02–1.23)
***PROM n (%)***			
Unadjusted OR (95% CI)	Ref	0.56 (0.48–0.67)*	0.31 (0.15–0.61)*
Adjusted OR (95% CI)	Ref	0.58 (0.48–0.70)*	0.37 (0.18–0.76)*
***Hyperthyroidism n (%)***			
Unadjusted OR (95% CI)	Ref	1.07 (0.66–1.74)	1.20 (0.28–3.15)
Adjusted OR (95% CI)	Ref	1.20 (0.70–2.05)	1.36 (0.30–4.08)
***Hypothyroidism n (%)***			
Unadjusted OR (95% CI)	Ref	0.93 (0.66–1.33)	1.49 (0.58–3.82)
Adjusted OR (95% CI)	Ref	0.92 (0.62–1.36)	1.39 (0.52–3.69)
***Caesarian n (%)***			
Unadjusted OR (95% CI)	Ref	1.59 (1.37–1.85)*	2.71 (1.67–4.39)*
Adjusted OR (95% CI)	Ref	1.01 (0.84–1.21)	1.61 (0.96–2.71)
***Neonatal Complications***			
***Preterm delivery *n* (%)***			
Unadjusted OR (95% CI)	Ref	0.89 (0.65–1.21)	0.63 (0.19–2.06)
Adjusted OR (95% CI)	Ref	1.11 (0.72–1.71)	0.79 (0.18–3.45)
***LGA n (%)***			
Unadjusted OR (95% CI)	Ref	0.41 (0.33–0.50)*	0.42 (0.20–0.90)*
Adjusted OR (95% CI)	Ref	0.46 (0.36–0.58)*	0.49 (0.23–1.05)
***SGA n (%)***			
Unadjusted OR (95% CI)	Ref	0.69 (0.59–0.82)*	0.42 (0.22–0.80)
Adjusted OR (95% CI)	Ref	1.12 (0.75–1.68)	1.20 (0.28–3.06)
***Macrosomia n (%)***			
Unadjusted OR (95% CI)	Ref	1.28 (0.94–1.75)	1.92 (0.85–4.36)
Adjusted OR (95% CI)	Ref	1.17 (0.81 - 1.70)	1.70 (0.72–4.01)
***Low birth weight n (%)***			
Unadjusted OR (95% CI)	Ref	0.73 (0.51–1.03)	0.74 (0.23–2.42)
Adjusted OR (95% CI)	Ref	0.84 (0.50–1.40)	0.83 (0.17–3.08)
***NICU n (%)***			
Unadjusted OR (95% CI)	Ref	0.58 (0.30–1.12)	2.49 (0.73–4.51)
Adjusted OR (95% CI)	Ref	0.56 (0.27–1.17)	1.62 (0.41–4.45)
***Neonatal Jaundice n (%)***			
Unadjusted OR (95% CI)	Ref	0.63 (0.53–0.74)*	0.36 (0.19–0.67)*
Adjusted OR (95% CI)	Ref	0.65 (0.44–0.95)*	0.40 (0.10–1.63)
***Neonatal Asphyxia *n* (%)***			
Unadjusted OR (95% CI)	Ref	0.68 (0.47–0.99)	0.99 (0.35–2.79)
Adjusted OR (95% CI)	Ref	0.63 (0.41–0.97)	0.99 (0.33–2.96)

PIH: Pregnancy induced hypertension; PROM: Premature rupture of membranes; LGA: Large for gestational age; SGA: Small for gestational age,

* p <0.05.

### Maternal Complications:

- Pregnancy-Induced Hypertension (PIH).
- Primiparous women had a lower risk of developing PIH than nulliparous women, with an adjusted odd ratio (OR) of 0.64 (95% CI, 0.45–0.92; *p* < 0.01).
- Multiparous women showed a lower risk reduction (not statistically significant) with an adjusted OR of 0.16 (95% CI, 0.02–1.23).

### Premature Rupture of Membranes (PROM):

Both primiparous and multiparous women had a significantly lower risk of PROM than nulliparous women, with adjusted ORs of 0.58 (95% CI, 0.48–0.70; p < 0.01) and 0.37 (95% CI, 0.18–0.76; *p* < 0.01), respectively.

### Caesarean Section:

Multiparous women exhibited a higher unadjusted risk of cesarean delivery (OR, 2.71; 95% CI, 1.67–4.39) than nulliparous women; however, statistical significance was lost after adjustment (adjusted OR, 1.61; 95% CI, 0.96–2.71).

### Neonatal Complications:

### Large for Gestational Age (LGA):

Primiparous women presented a lower risk of having an LGA infant, with an adjusted OR of 0.46 (95% CI, 0.36–0.58; *p* < 0.01) than nulliparous women, whereas multiparous women had an adjusted OR of 0.49 (95% CI, 0.23–1.05).

### Neonatal Jaundice:

The risk of neonatal jaundice was lower in primiparous mothers (adjusted OR, 0.65; 95% CI, 0.44–0.95; *p* < 0.05) than in nulliparous women. Multiparous women also showed a low risk (adjusted OR, 0.40; 95% CI, 0.10–1.63), without statistical significance.

### Neonatal Asphyxia:

Primiparous women had a significantly reduced risk of neonatal asphyxia than nulliparous women (adjusted OR, 0.63; 95% CI, 0.41–0.97; *p* < 0.05).

## DISCUSSION

Our primary aim with this study was to identify associations between parity, GWG, and pregnancy outcomes in women with GDM. We hypothesized that parity would have a significant impact on the GWG and subsequent maternal and neonatal outcomes. Our findings ruled out the null hypothesis, revealing that higher parity is associated with distinct patterns of GWG and varying risks of adverse outcomes.

The study’s results reveal significant associations between parity and various neonatal and maternal outcomes among women with GDM. Demographic and GWG characteristics varied across the nulliparous, primiparous, and multiparous groups. Notably, primiparous women were slightly older, having a large representation particularly in the 30-40 age group, and they had higher BMI values, particularly among those classified as overweight or obese. Gestational weights at different pregnancy stages were consistently higher in primiparous women than in nulliparous and multiparous women, with significant differences observed at all measured time points. This trend was also reflected in total GWGs and weight gains at specific gestational weeks, where nulliparous women generally gained more weight than their primiparous and multiparous counterparts. As regards maternal complications, primiparous women exhibited a lower adjusted risk of developing PIH compared to nulliparous women, with multiparous women showing an even greater risk reduction (without statistical significance). Nulliparous women had a significantly higher risk of PROM than either primiparous or multiparous women. However, multiparous women had a higher unadjusted risk of requiring a caesarean section, although this risk was not significant after adjusting for confounders. In terms of neonatal outcomes, primiparous women had a lower risk of delivering a LGA infant, and this trend was similar in multiparous women, although less pronounced. Additionally, neonatal jaundice risks were significantly lower in both primiparous and multiparous women compared to the risk in nulliparous women. Primiparous women also exhibited a significantly reduced risk of neonatal asphyxia.

These findings suggest that parity influences neonatal and maternal outcomes in pregnancies complicated by GDM, with higher parity generally being associated with a reduction in certain risks such as those of PIH, PROM, neonatal jaundice, and asphyxia. However, higher parity may increase the likelihood of cesarean delivery. These insights underscore the importance of considering parity when managing pregnancies in women with GDM to optimize maternal and neonatal health outcomes.

GDM is a significant public health concern that affects a substantial proportion of pregnant women worldwide.^22^ Its management is crucial to prevent adverse neonatal and maternal outcomes. Among the various factors influencing pregnancy outcomes in women with GDM, parity and GWG stand out as critical determinants.^23^,^24^ Parity is defined as the number of previous pregnancies a woman has carried to a viable gestational age.^25^ Understanding how parity and GWG interact and affect pregnancy outcomes is important, particularly in the context of GDM, where the stakes for both mother and child are high.^25^

These findings are also consistent with those in other studies^11^,^12^ that have shown an inverse relationship between parity and certain pregnancy complications. For example, multiparous women have been reported to have lower rates of PIH and PROM than their nulliparous counterparts.^26^,^27^ By contrast, our study results differ from earlier research in a few key areas. For instance, many studies have primarily focused on the effects of parity on general obstetric outcomes^28^, whereas our research specifically examined its impact in the context of GDM, a high-risk condition that requires specialized care. Additionally, we comprehensively analyzed GWGs across different parity groups, highlighting the nuanced ways in which parity influences weight gains during pregnancy and the subsequent risks of adverse outcomes.

The consistency of our findings with those of previous research suggests that the association between parity and pregnancy outcomes in GDM may be influenced by several underlying mechanisms. The physiological adaptations that occur with each successive pregnancy may have a substantial role in these mechanisms.^29^-^32^ Multiparous women may experience more efficient metabolic regulation and less inflammatory responses during pregnancy, factors which could account for their lower risk of complications like PIH and PROM.^33^ Additionally, the experience of previous pregnancies might lead to more effective maternal behaviors and lifestyle adjustments, contributing to healthier pregnancy outcomes.^34^ To ensure the robustness of our results, we accounted for several potential confounders, including maternal age, BMI, GWG, and gestational weight at delivery. By adjusting for these variables, we aimed to isolate the effect of parity on pregnancy outcomes as accurately as possible.

### Limitations:

The retrospective design may have introduced recall biases, particularly in self-reported pre-pregnancy weight and lifestyle factors that may have influenced GWGs.^35^ Moreover, the relatively small number of multiparous women limits the generalizability of our findings to this group. A notable limitation of our study is the absence of data on additional indicators, such as the length of hospital stay and delivery time, which are important metrics for assessing the overall maternal and neonatal healthcare burden. Including these variables in future studies could provide a more comprehensive understanding of how parity and GWG influence not only clinical outcomes but also healthcare utilization and recovery dynamics.

### Strength of the study:

Despite its limitations, our study possesses crucial strengths. The strengths of this study include its large sample size, detailed data collection from a comprehensive hospital database, and robust statistical methods to adjust for potential confounders such as maternal age, pre-pregnancy BMI, and gestational age at delivery. The use of a well-defined cohort of women with GDM ensures the reliability and relevance of the findings. The large, well-defined cohort of women with GDM provided a rich dataset for examining the impact of parity and GWG on pregnancy outcomes. The use of data from a comprehensive hospital database ensures that their reliability and completeness, minimizing the risk of missing information. Moreover, insights from our study could provide clinical guidelines and interventions aimed at improving outcomes for women with GDM.

### Future perspective:

Despite these strengths, there are areas that require further investigation. The mechanisms underlying the observed relationships between parity, GWG, and adverse outcomes remain unclear and warrant exploration in prospective studies. For instance, physiological adaptations with successive pregnancies and behavioral differences between parity groups may play a role and should be investigated. Looking forward, future research should validate our findings in larger, more diverse populations.

Prospective studies with more detailed data on lifestyle factors, dietary habits, and physical activity during pregnancy could help elucidate the mechanisms behind the observed associations. Future research should also evaluate the long-term effects of parity and GWG on maternal and offspring health, particularly the risk of Type-2 diabetes and obesity. Additionally, investigating the role of specific interventions, such as tailored nutritional and exercise programs for women with different parity levels, could provide actionable strategies for managing GWG thereby reducing the risk of adverse outcomes in GDM.^36^

## CONCLUSION

The findings of this study underscore the critical need for parity-specific guidelines in the management of GDM. Parity significantly influences GWG and is associated with varying risks of maternal and neonatal complications, suggesting that a one-size-fits-all approach may not be optimal for this population. Developing guidelines that account for the interplay between parity, GWG, and pregnancy outcomes can enable clinicians to provide targeted care, such as tailored nutritional counseling, weight monitoring, and early interventions for high-risk groups. For instance, nulliparous women, who may be at higher risk for complications such as PIH and PROM, might benefit from more rigorous monitoring and preventive strategies. In contrast, multiparous women, who demonstrate unique patterns of GWG and reduced risks for certain complications, may require different management approaches to optimize outcomes. By incorporating parity as a key factor in clinical guidelines, healthcare providers can implement interventions that are both targeted and effective, ultimately improving the health and well-being of women with GDM and their infants. This parity-specific approach has the potential to reduce adverse outcomes, enhance maternal and neonatal health, and guide future research and policy in obstetric care.

### Authors’ contributions:

**LL:** Study design, literature search and manuscript writing.

**RC**, **HL**, **TY**, **ZC** and **LZ:** data collection, analysis interpretation and critical review.

**LL:** Was involved in the manuscript revision and validation. and is responsible for the integrity of the study.

All authors have read, approved the final manuscript and are accountable for the integrity of the study.
